# Type III Osteogenesis Imperfecta and *COL1A1* Pathological Variants Are Associated With Higher Incidence and Progression of Hearing Loss

**DOI:** 10.1155/humu/6245371

**Published:** 2026-08-21

**Authors:** Julie A. Christensen, Anne Tran, Hui Cheng, Sara Talvacchio, Carmen C. Brewer, Christopher Zalewski, Jennifer Chisholm, Talah T. Wafa, L. Noelle Allemang, Elena F. Evans, Alberta Derkyi, Joan C. Marini, Gayla L. Poling

**Affiliations:** ^1^ Auditory and Vestibular Clinical Research Section, Neurotology Branch, National Institute on Deafness and Other Communication Disorders (NIDCD), National Institutes of Health, Bethesda, Maryland, USA, nih.gov; ^2^ Data Science Core, National Institute on Deafness and Other Communication Disorders (NIDCD), National Institutes of Health, Bethesda, Maryland, USA, nih.gov; ^3^ Office of the Clinical Director, Division of Intramural Research, Eunice Kennedy Shriver National Institute of Child Health and Human Development (NICHD), National Institutes of Health, Bethesda, Maryland, USA, nih.gov; ^4^ Section on Heritable Disorders of Bone and Extracellular Matrix, Division of Intramural Research, Eunice Kennedy Shriver National Institute of Child Health and Human Development (NICHD), National Institutes of Health, Bethesda, Maryland, USA, nih.gov

## Abstract

**Purpose:**

This study interrogates longitudinal natural history data to determine whether hearing loss (HL) incidence, onset, degree, and type in osteogenesis imperfecta (OI) caused by heterozygous collagen missense variants are associated with a specific gene or combinations of causal gene and OI type.

**Methods:**

Audiological evaluation was conducted in 73 children with Type III or IV OI carrying *COL1A1* or *COL1A2* missense, exon‐skipping, or small deletion variants, with adult follow‐up.

**Results:**

A total of 71% of Type III OI participants experienced HL, compared with 39% of those with Type IV OI. Conductive HL persisted (33%) or transitioned to mixed conductive/sensorineural (67%) with progression. While HL was predominantly mild in most participants, six Type III participants (25%) but only one Type IV OI participant (7%) progressed to more severe loss. Most Type III and IV OI participants had HL onset in the first decade of life, with 46% and 33% of onsets, respectively, occurring before school age. More than half of participants with *COL1A1* or *COL1A2* variants had HL. Interfamilial and intrafamilial variability in HL was noted. Statistical interaction between genotype and phenotype indicated the greatest HL progression among Type III OI with *COL1A1* variants.

**Conclusions:**

Our data reveal significant HL onset in the first decade, and an interaction of Type III OI and COL1A1 variants among those with HL progression. These findings will guide clinical management, facilitating early monitoring tailored to genotype and phenotype.

## 1. Introduction

Osteogenesis imperfecta (OI) is a connective tissue disorder primarily caused by heterozygous variants in Type I collagen and characterized by bone fragility, skeletal deformities, and short stature [1]. OI patients with deleterious collagen variants are classified phenotypically by the Sillence classification [2], in which OI Type II is perinatal lethal, OI Type III is severe progressive deforming, and OI Type IV is moderately‐severe, whereas Type I OI is mild. The dominant, recessive, and X‐linked OI types identified subsequently, primarily by genetic etiology, comprise Types V–XXII [1, 3]. In addition to fragility and deformity of the axial skeleton, patients with OI may display a variety of secondary connective tissue features, including blue sclerae, dentinogenesis imperfecta, impaired hearing, and cardiopulmonary impairment [3].

Hearing loss (HL) has been reported to impact people with OI with an overall prevalence ranging from 28% to 66% [4–9]. In the pediatric population, the prevalence is reportedly lower, 0%–25% [5, 10–13]. The degree of HL in OI is typically mild [6, 9], but can be moderate, severe, or profound. It usually begins as conductive hearing loss (CHL) that progresses to mixed hearing loss (MHL) or sensorineural hearing loss (SNHL) with aging [5, 12, 14, 15], although SNHL is reported to occur at all ages [4, 8, 11, 15, 16]. The majority of HL in OI is described as bilateral and symmetrical for type and degree [8, 14]. Studies of HL in OI are starting to emerge beyond Europe and North America. In 2015, Lin et al. [17] associated HL with helical mutations (17%) and haploinsufficiency (21%) in a Taiwanese OI population.

Machol et al. [9] recently reported on HL in OI in a large cross‐sectional population of North American participants in the Brittle Bone Research Consortium (BBRC). In contrast to studies predominantly reporting HL as beginning in the second or third decade of life [5, 6, 14, 15, 18], the BBRC [9] reported HL beginning in the first decade of life for approximately 20% of participants with OI Type III. This rate of childhood onset is higher than reported for a European OI population with known collagen‐based OI, which found 6.3% of total ears experienced HL onset before the age of 10 years [18]. The BBRC [9] also found a higher prevalence of SNHL in females at all ages, although this difference was not significant in individuals younger than 30 years of age. A 2019 literature review by Carre et al. [14], reported no gender differences in the prevalence of HL.

Heterozygous variants in the genes encoding Type I collagen, *COL1A1* and *COL1A2*, are responsible for 80%–90% of OI cases reported in North America [19]. Studies on the relationship between gene variants and HL are limited. In 1990, Sykes et al. [20] reported “presenile hearing loss” restricted to individuals with a *COL1A1* variant in the United Kingdom. In 2004, Hartikka et al. [7] genotyped a Finnish OI population, mean age 36.4 years, among which 17 (about one‐third) had null *COL1A1* variants diagnostic for OI Type I. Among 21 patients with *COL1A1* and 11 with *COL1A2* heterozygous missense or exon skipping variants, they found no correlation between hearing status and mutated gene. In 2011, Swinnen et al. [18] examined the correlation between HL and the underlying variant in 114 OI patients (64 families from Belgium, Netherlands, and Italy) who had developed HL before the age of 40 or had normal hearing after the age of 40. In this group, two‐thirds of patients had null variants in one *COL1A1* allele, indicative of OI Type I, whereas the remaining patients had heterozygous missense or exon skipping variants in *COL1A1*/*COL1A2*. Again, a correlation between HL and mutated gene or variant type was not found.

Here, we report on audiological findings among 73 participants in the Longitudinal OI Natural History Study of the National Institute of Child Health and Human Development (NICHD) at the NIH Clinical Center, Maryland, United States. Patients were enrolled as children and received repeat audiological evaluations and follow‐up as adults. All patients had heterozygous variants altering the primary protein sequence encoded by *COL1A1* or *COL1A2* as the etiology of OI Type III or IV. The goals of this study were to: (1) determine whether patients with missense/exon skipping variants in a specific collagen gene or combinations of causal gene and OI type experience HL; (2) leverage longitudinal data to identify patterns in progression of HL by type and degree in relation to gene variant and OI type; and (3) identify age of HL onset.

## 2. Materials and Methods

### 2.1. Study Population

Longitudinal audiometric evaluations obtained between 1980 and 2023 were analyzed on 75 participants diagnosed with OI Type III or IV with heterozygous variants in *COL1A1* or *COL1A2* (Table 1). All participants had confirmed heterozygous variants in *COL1A1* or *COL1A2* resulting in small changes in the primary structure of the collagen alpha chains. The majority were missense substitutions for glycine residues; two were single exon skipping, and one was a small deletion. Mutations were determined by targeted gene panel sequencing (CTGT [now HNL Genomics], Allentown, Pennsylvania) with a few exceptions. Causative mutations for ID 208, 125, and 121 were reported by the Marini lab in 1993, 1996, and 2002, respectively [21–23]. Their cDNA level mutations and any intronic changes were sequenced by the dideoxy chain‐termination method (Sanger sequencing) using appropriate PCR amplification products.

**Table 1:** Demographics of participants included in the study^a^.

Variables	*COL1A1*	*COL1A2*	Total
	(*n* = 41)	(*n* = 32)	(*n* = 73)
Sex, *n* (%)			
Female	20 (48.8)	21 (65.6)	41 (56.2)
Male	21 (51.2)	11 (34.4)	32 (43.8)
OI type, *n* (%)			
OI Type III	22 (53.7)	12 (37.5)	34 (45.2)
OI Type IV	19 (46.3)	20 (62.5)	39 (52.1)
Age at assessment, (years)			
Minimum	1	2	1
Maximum	40	45	45
Mean ± SD	15.1 ± 8.8	14.1 ± 8.6	14.7 ± 8.7
Visits			
Minimum	1	2	1
Maximum	18	20	20
Mean ± SD	7.8 ± 4.7	7.2 ± 4.5	7.5 ± 4.6

^a^Race and ethnicity self‐identified and obtained via electronic medical record as: White and not Hispanic or Latino (*n* = 60), Black and not Hispanic or Latino (*n* = 7), Hispanic or Latino with race marked as “unknown” (*n* = 4), Asian (*n* = 2), and not Hispanic or Latino with race marked as “unknown” (2). Additional information on identity and ancestry was self‐reported by participants in the medical record via the clinician as follows: Jewish (*n* = 6), Bulgarian (*n* = 2), Italian (*n* = 2), Caribbean (*n* = 1), Chilean (*n* = 1), Chinese (*n* = 1), German (*n* = 1), Guatemalan (*n* = 1), Middle Eastern (*n* = 1), and Vietnamese (*n* = 1).

The legacy notation for collagen protein variants, in which the first Gly of the collagen helical region is designated as Amino Acid 1 [18], is utilized in descriptions throughout this text; the current ACMG notation for their variants is also presented in Table 2. Children with OI from throughout the United States enrolled in a natural history study (Clinical Trials NCT03575221; Natural History of the Collagen‐Related Disorder OI and Genotype–Phenotype Correlation and NCT00001594; Evaluation and Intervention for the Effects of OI) approved by the institutional review board. All participants or their guardians gave informed consent. Participants were seen throughout childhood, with periodic examination of the primary skeletal and secondary audiological, dental, pulmonary, and neurological features of OI. Of the 75 participants, one was excluded from analysis because ear‐specific responses could not be obtained, and one was excluded because no pure tone averages (PTAs) could be calculated from the limited number of frequencies tested.

**Table 2:** Characteristics of individuals with hearing loss (*n* = 39).

ID	Gene	Legacy notation	ACMG DNA notation	ACMG protein notation	OI type	Sex	Min age	Max age	Max degree HL	HL types	LF‐PTA degree HL	4F‐PTA degree HL	HF‐PTA degree HL
128	*COL1A1*	G76E	c.761G>A	p.G254E	III	F	8	19	Profound∗	CHL/MHL	B(profound)	B(profound)	B(profound)
131	*COL1A1*	G88E	c.797G>A	p.G266E	III	F	9	29	Severe∗	CHL/MHL	B(severe)	B(mod)	B(mod)
111	*COL1A1*	G148D	c.977G>A	p.G326D	IV	M	7	22	Mild	CHL/SNHL	B(mild)	L(mild)	L(mild)
142	*COL1A1*	G154R	c.994G>A	p.G332R	III	M	4	26	Mild	CHL/SNHL	L(mild)	L(mild)	L(mild)
124	*COL1A1*	G187A	c.1094C>A	p.G365A	III	F	6	27	Mild	CHL/SNHL	B(mild)	—	—
130	*COL1A1*	G193S	c.1111G>A	p.G371S	III	F	4	15	Mild	CHL	B(mild)	—	B(mild)
102	*COL1A1*	G217S	c.1183G>A	p.G395S	III	F	11	31	Mild	SNHL	R(mild)	—	—
107	*COL1A1*	G286R	c.1390G>A	p.G464R	III	M	10	32	Severe∗	CHL/MHL	R(severe)	R(severe)	B(severe)
											L(mod)	L(mod)	
133	*COL1A1*	G352S	c.1588G>A	p.G530S	IV	M	7	39	Mild	CHL/SNHL	B(mild)	—	—
134	*COL1A1*	G352S	c.1588G>A	p.G530S	IV	M	4	36	Mild	CND	R(mild)	—	L(mild)
139	*COL1A1*	G352S	c.1588G>A	p.G530S	IV	F	3	22	Mild	CHL	B(mild)	R(mild)	—
125	*COL1A1*	Splice out exon 33‐36	c.2337_2451+62del g.50190267_50190825del		III	F	3	23	Mild	CHL	B(mild)	B(mild)	—
119	*COL1A1*	G589S	c.2299G>A	p.G767S	III	F	2	17	Mild	CHL	B(mild)	—	—
136	*COL1A1*	G589S	c.2299G>A	p.G767S	III	M	3	30	Mild	CHL/SNHL	B(mild)	B(mild)	B(mild)
141	*COL1A1*	G613A	c.2371G>A	p.G791A	III	M	6	10	Mild	CHL	R(mild)	—	—
121	*COL1A1*	Splice out exon 41	g.12743A>C	IVS41+4A>C	III	M	3	15	Mild	CND	L(mild)	—	—
135	*COL1A1*	G832S	c.3028G>A	p.G1010S	IV	F	2	40	Severe(R)∗	CHL/MHL(R)	R(severe)	R(mod)	R(mod)
									Mild(L)	CHL/SNHL(L)	L(mild)	L(mild)	L(mild)
110	*COL1A1*	G898S	c.3226G>A	p.G1076S	III	F	4	22	Severe∗	CHL/MHL	R(severe)	B(mod)	R(mod)
											L(mod)		L(severe)
118	*COL1A1*	G898S	c.3226G>A	p.G1076S	III	M	5	10	Mod∗	CHL	B(mod)	R(mod)	R(mild)
												L(mild)	
127	*COL1A1*	G997S	c.3523G>A	p.G1175S	III	M	4	28	Mod(R)∗	CHL	R(mod)	R(mod)	R(mod)
									Mild(L)		L(mild)	L(mild)	L(mild)
132	*COL1A1*	P1266H CPRO peptide	c.4331C>A	p.P1444H	IV	F	3	9	Mild	CND	B(mild)	—	—
207	*COL1A2*	G106V	c.589G>T	p.G196V	IV	F	2	36	Mild	CHL/SNHL	L(mild)	L(mild)	L(mild)
208	*COL1A2*	Splice out exon 16	g.16194G>A	IVS16+1G>A	IV	F	4	33	Mod(L)	CHL	L(mild)	L(mod)	R(mild)
									Mild(R)				L(mod)
203	*COL1A2*	G190V	c.839G>T	p.G280V	III	F	4	22	Mild	CHL	R(mild)	R(mild)	—
212	*COL1A2*	G238S	c.982G>A	p.G328S	IV	F	2	35	Mild	CHL	L(mild)	—	—
224	*COL1A2*	G238S	c.982G>A	p.G328S	III	F	3	6	Mild	CND	B(mild)	—	—
223	*COL1A2*	G247C	c.1009G>T	p.G337C	III	M	17	26	Mild	CHL/CND	B(mild)	R(mild)	—
202	*COL1A2*	G247S	c.1009G>A	p.G337S	IV	M	8	45	Mild	CND	—	—	B(mild)
219	*COL1A2*	G250S	c.1018G>A	p.G340S	III	F	6	16	Mild	CHL	B(mild)	—	R(mild)
211	*COL1A2*	G268S	c.1072G>A	p.G358S	IV	F	6	26	Mild	CHL	R(mild)	—	—
200	*COL1A2*	G337S	c.1279G>A	p.G427S	III	F	7	18	Mild	CHL/SNHL	B(mild)	—	—
222	*COL1A2*	G370S	c.1378G>A	p.G460S	III	M	4	12	Mild	CND	—	—	L(mild)
213	*COL1A2*	G511S	c.1801G>A	p.G611S	IV	F	14	35	Mild	CND	—	—	B(mild)
218	*COL1A2*	G703R	c.2377G>A	p.G793R	III	F	3	14	Mild	CHL/CND	B(mild)	—	—
221	*COL1A2*	G706S	c.2386G>A	p.G796S	III	F	4	5	Mild	CND	B(mild)	—	—
220	*COL1A2*	G922S	c.3034G>A	p.G1012S	III	F	5	17	Mild	CHL	B(mild)	R(mild)	B(mild)
228	*COL1A2*	G922S	c.3034G>A	p.G1012S	IV	F	25	28	Mild	CHL/SNHL	R(mild)	B(mild)	B(mild)
201	*COL1A2*	G940D	c.3089G>A	p.G1030D	IV	M	8	22	Mild	CHL	R(mild)	—	—
120	*COL1A2*	P1011L	c.3299C>T	p.P1101L	IV	M	3	18	Mild	CND	—	L(mild)	—

*Note:* All columns describing hearing loss report maximum degree. Asterisk “∗” denotes progressive hearing loss.

Abbreviations: 4F‐PTA, frequency pure tone average; B, both ears; CHL, conductive hearing loss; F, female; HF‐PTA, high frequency pure tone average; HL, hearing loss; ID, identifying number; L, left ear; LF‐PTA, low frequency pure tone average; M, male; mod, moderate; MHL, mixed hearing loss; R, right ear; SNHL, sensorineural hearing loss.

Primary outcomes included HL type, degree, age of onset, and progression. Secondary outcomes included “breakpoints” in trajectory of progressive HL. Sex was defined as sex documented in the medical record and collected to determine prevalence of sex‐specific audiologic manifestations.

### 2.2. Defining Hearing Status

Comprehensive, age‐appropriate audiometric evaluations were completed in sound‐treated booths and consisted of behavioral audiometry for air conduction (AC) from 0.25 to 8 kHz and bone conduction (BC) from 0.25 to 4 kHz, speech reception thresholds, word recognition ability in quiet, 226‐Hz tympanometry, and acoustic reflex threshold and decay testing. A subset of these tests was used for analysis. Hearing status was evaluated using three PTAs: 4‐frequency (4F) PTA (0.5, 1, 2, and 4 kHz); low frequency (LF) PTA (0.25 and 0.5 kHz); and high frequency (HF) PTA (4 and 8 kHz) (see Table S1 for detailed definitions in this section).

HL was defined in individual ears by the identification of PTAs > 20 dBHL in any of the three PTAs at one or more visits. HL was characterized based on degree, type, age of onset, and progression. HL type was determined as: (1) CHL defined as the presence of an air‐bone gap (ABG) > 10 dB when AC > 20 dBHL and BC ≤ 20 dBHL; (2) MHL defined as AC and BC > 20 dBHL in combination with ABG > 10 dBHL; and (3) SNHL defined as AC > 20 dBHL with ABG ≤ 10 dBHL.

Degree of HL was defined as mild (> 20 and ≤ 40 dBHL); moderate (> 40 and ≤ 70 dBHL); severe (> 70 and ≤ 95 dBHL); or profound (> 95 dBHL). Hearing sensitivity was defined as normal if all three PTAs were ≤ 20 dBHL. This cutoff was chosen to establish a balance between the normal hearing thresholds for children (15 dBHL) and adults (25 dBHL) [24, 25]. HL was defined as stable unless there was progression. Progressive HL was defined as worsening beyond mild into a greater degree for the duration of the time the participant was followed. In audiometry, test–retest variability of ±5 dBHL is expected and does not demonstrate a change in hearing sensitivity [26].

### 2.3. Statistical Analysis

Statistical analyses were performed in R software (Version 4.3.3). LF‐PTA, 4F‐PTA, and HF‐PTA were log‐transformed to address nonnormality of residuals and heteroscedasticity [27] (i.e., nonconstant variance of residuals across levels of the fitted values). Linear mixed‐effects models were fitted using the *lme4* package (v1.1.34) to examine association between log‐transformed PTA outcomes and fixed effects, including *genotype* (*COL1A1* vs. *COL1A2*), *OI type* (III vs. IV), and *Visit* time (modeled continuously). *Sex* and *age* at onset of HL were included as covariates. All main effects and interaction terms among *visit*, *genotype*, and *OI type* were included to assess both independent and joint effects of genetic background, clinical subtype, and longitudinal progression on hearing thresholds. The model was specified as follows:

logPTA~sex+age+visit×genotype×OI type+1subject/ear.

Random intercepts were included for subjects and ears nested within subjects, to account for repeated measurements within individuals and between ears. Model fit was evaluated using Akaike information criterion and Bayesian information criterion [28], which balance model goodness‐of‐fit against model complexity, with lower values indicating a more parsimonious model. Model assumptions were assessed using diagnostic plots generated with the plot_model() function (type = “diag”) from the *sjPlot* package (v2.8.14).

Statistical significance of fixed effects and interaction terms was assessed using *F*‐tests with degree of freedom estimated via Satterthwaite′s approximation [29], which provides improved small‐sample inference for mixed‐effects models by approximating denominator degrees of freedom, implemented in the *lmerTest* package. Post hoc contrasts and visit‐related slopes were estimated from the linear mixed‐effects model adjusting for age and sex, with random intercepts for subject and ear. Positive slopes indicate increasing PTA values (i.e., worsening hearing thresholds) over time (Table S2). Post hoc pairwise comparisons and slope analyses were conducted using the *emmeans* package (v1.10.1), with Tukey adjustment applied for multiple comparisons.

To further characterize HL progression, the ages of inflection breakpoints were quantified. To identify breakpoints, we applied segmented linear regression to AC PTA data for the individual ears of the seven patients with progressive HL. This approach models the PTA trajectory as a series of linear segments separated by breakpoints, allowing detection of time points at which the rate of hearing change (slope) significantly shifts. Analyses were conducted using the *segmented* package (v2.2‐1) in R.

## 3. Results

### 3.1. Study Population and Audiometric Evaluation

The final study population included 73 participants (Table 1), of whom 41 had *COL1A1* variants (female/male = 20/21; OI Type III/IV = 22/19), and 32 had *COL1A2* variants (female/male = 21/11; OI Type III/IV = 12/20). Audiological evaluations were conducted on individuals aged 1–45 years (mean age [years ± SD]: OI Type III 13.5 ± 7.6; OI Type IV 15.7 ± 9.5; *COL1A1* 15.1 ± 8.8; *COL1A2* 14.1 ± 8.6). Participants underwent between 1 (*n* = 3) and 20 (*n* = 1) hearing tests, with means of 7.8 ± 4.7 and 7.2 ± 4.5 tests per individuals for participants with *COL1A1* and *COL1A2* variants, respectively.

### 3.2. Audiometric Findings by OI Type

#### 3.2.1. Incidence of HL

A total of 53% (*n* = 39) of the 73 total participants with OI Type III or IV exhibited HL (Table 2). For OI Type III, 71% (n = 24/34) of individuals had HL, compared with 38% (*n* = 15/39) in OI Type IV. Among the individuals with HL, the proportions of unilateral and bilateral HL were comparable between OI Type III (*n* = 6/24, 25% and *n* = 18/24, 75%, respectively) and OI Type IV (*n* = 6/15, 40% and *n* = 9/15, 60%, respectively) (Figure S1).

#### 3.2.2. Degree of HL and Progression

**Figure 1:**
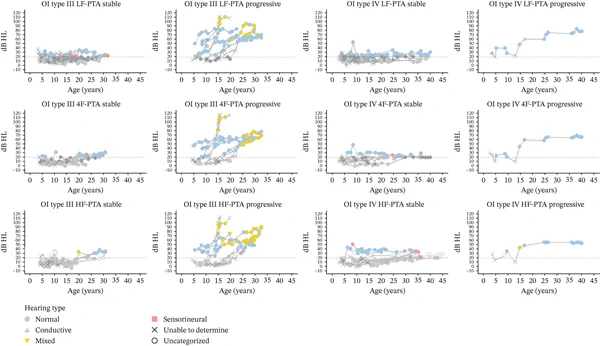
Degree of hearing loss and progression by OI type. All ears (individual lines) with hearing loss (horizontal dashed line represents degree of hearing loss designation; normal hearing = gray circle) and type (CHL = blue triangle, MHL = yellow inverted triangle, and SNHL = red square) indicated as a function of age (years). Each row represents LF‐, 4F‐, and HF‐PTA with columns noting stable or progressive hearing loss, by OI Type III or IV. Bone conduction not measured indicated by “x,” and unfilled circles represent cases unable to be categorized in terms of hearing loss type. Refer to Figure S1 for representation of all ears in the study (including normal hearing participants).

Most affected participants had mild HL (75% OI Type III and 93% OI Type IV). Six participants with OI Type III experienced greater HL, ranging from moderate (*n* = 2, 8%) to severe (*n* = 3, 13%) to profound HL (*n* = 1, 4%). For OI Type IV, only one participant had HL that progressed, developing severe loss in one ear (Figure 1).

#### 3.2.3. Type of HL and Progression

Among individuals with HL, 25% (*n* = 6/24) of those with OI Type III experienced a progressive decline, compared with 7% (*n* = 1/15) of those with OI Type IV. In OI Type III, progressive HL either remained conductive (33%) or transitioned from conductive to MHL (67%). The single progressive HL in OI Type IV remained conductive over a period of 37 years. Notably, the individual with OI Type IV and one individual with OI Type III demonstrated bilateral HL that was stable in one ear and progressive in the other. For all other individuals with stable HL, 50% of those with OI Type III and 39% of those with OI Type IV experienced mild CHL. A smaller subset, 22% in OI Type III and 23% in OI Type IV, transitioned from mild CHL to mild SNHL. Six percent of individuals with OI Type III and 8% of those with OI Type IV had SNHL with no history of CHL or MHL. Among those with stable HL, the type of loss could not be classified in 22% of OI Type III and 31% of OI Type IV (Figure 1).

#### 3.2.4. Age of Onset of HL

**Figure 2:**
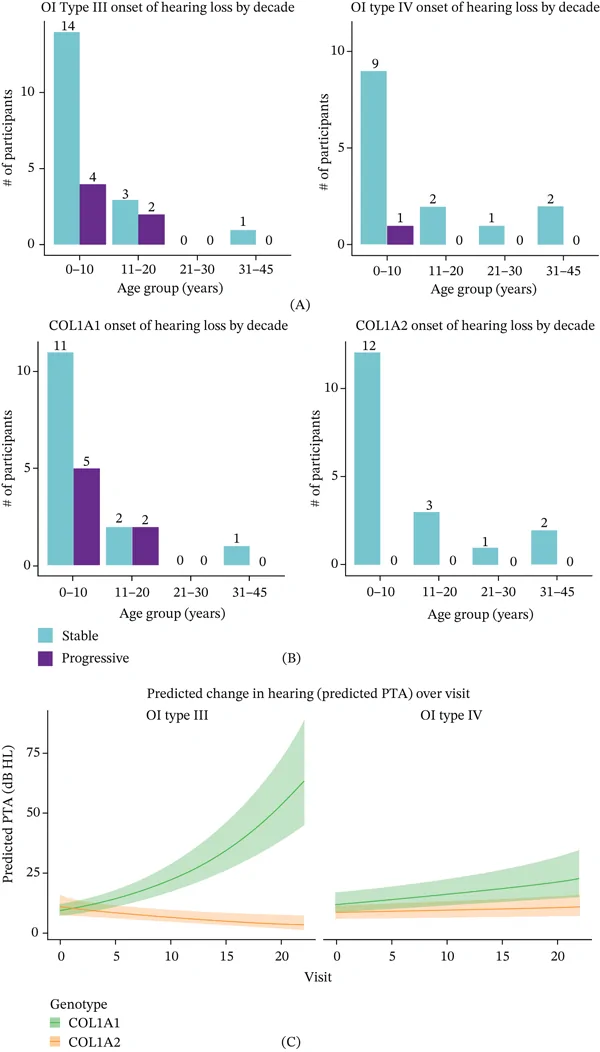
Hearing loss onset and progression. Stable (filled light blue bars) and progressive (filled dark purple bars) hearing loss represented by decade for age group in years. (A) Hearing loss onset in OI Types III and IV occurs most commonly in first decade of life (*n* = 18 OI Type III; *n* = 10 OI Type IV). (B) Hearing loss onset in *COL1A1* and *COL1A2* occurs most commonly in the first 5 years of age (*n* = 10; *n* = 16 first decade) in *COL1A1* and in the first decade (*n* = 12; *n* = 6 first 5 years) for *COL1A2*. (C) *COL1A1* variants (in green) showed increased hearing threshold indicating greater hearing loss and steeper progression over time in OI Type III (left panel) versus OI Type IV (right panel) and *COL1A2* variants (in orange), and OI Type III showed a slight decrease in hearing threshold over time (visit). Visit slope: *COL1A1*, OI Type III, *β* = 0.038, *p* = <0.0001; *COL1A2*, OI Type III, *β* = −0.023, *p* = 0.0023; *COL1A1*, OI Type IV, *β* = 0.013, *p* = 0.0014; *COL1A2*, OI Type IV, *β* = 0.0048, *p* = 0.14.

For both OI Types III and IV, the age of HL onset was earlier than previously reported in most studies, with the majority occurring in the first decade of life (*n* = 18/24 [75%] OI Type III; *n* = 10/15 [67%] OI Type IV) (Figure 2A). First decade onset occurred among individuals with both stable and progressive HL. Among individuals with stable HL, 78% (*n* = 14/18) of those with OI Type III and 87% (*n* = 13/15) of those with OI Type IV experienced onset within the first decade. Among individuals with progressive HL, 67% (*n* = 4/6) of individuals with OI Type III and the one individual with OI Type IV had onset within the first decade. In the first 5 years of the first decade, HL onset was 46% (*n* = 11/24) in OI Type III and 33% (*n* = 5/15) in OI Type IV. In later decades, HL onset occurred in 21% (*n* = 5/24) and 13% (*n* = 2/15) during the second decade, 0% and 7% (*n* = 1/15) during the third decade, and 4% (*n* = 1/24) and 13% (*n* = 2/15) in the fourth and fifth decades for individuals with OI Types III and IV, respectively. This may be limited by an examination of fewer patients past their third decade of life (Table S3).

### 3.3. HL and Genotype–Phenotype Correlation

#### 3.3.1. HL and Correlation With Alpha Chain Position of Missense Variant

More than half of participants with *COL1A1* (*n* = 21/41; 51%) or *COL1A2* (*n* = 18/32; 56%) variants had HL (Table 2). Except for a few individuals, the participants had missense variants, mostly glycine substitutions. Several participants had exon splicing defects or a large deletion. There was no correlation of splicing defect versus glycine substitution with HL. Our population did not include individuals with *COL1A1* haploinsufficiency defects causing Type I OI.

**Figure 3:**
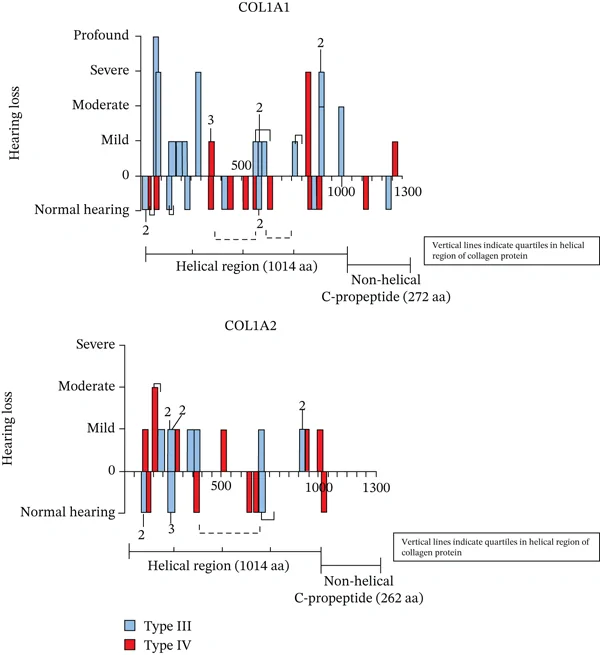
Hearing loss map. Normal hearing compared with hearing loss (by degree on *y*‐axis) mapped onto Type I collagen amino acid chain for OI Type III (blue hashed fill) and OI Type IV (red solid fill) participants with *COL1A1* (top panel) and *COL1A2* (bottom panel) variants. Each filled bar represents a single amino acid substitution. Bars above the baseline indicate hearing loss by degree, and bars below the baseline indicate normal hearing. Each bar represents an individual patient unless there is a number next to the bar indicating multiple patients with the same variant and audiology finding. Splicing mutations or deletions are indicated with brackets spanning the amino acids affected by the exon splice. Below the HL map, there are two additional sets of brackets. First, in the regions indicated by dashed lines, OI variants are associated with normal hearing. In our OI cohort, no or few patients with variants in this region have HL. Second, brackets composed of solid lines indicate the helical and C‐terminal nonhelical portions of the collagen alpha chains. Quartiles along the helical region are indicated by vertical lines.

Figure 3 shows the collagen variant position of individual participants with and without HL along the pro*α*1(I) and pro*α*2(I) chains. HL is associated with heterozygous collagen protein sequence variants along the full length of the pro*α* chains, including the C‐propeptides. In the helical region of the *α*1(I) chain, there are two substantial gaps on the HL map, from Gly352 to Gly589 and from Gly613 to Gly832, representing approximately one‐half the length of the chain. Some participants with missense variants in these regions have normal hearing. In the *α*2(I) chain map, there is only one individual with HL in the region from Gly370 to Gly703. In addition, for individuals with OI Type IV, we note a lower incidence of HL in the third quartile of the Type I collagen helix with either *COL1A1* or *COL1A2* variants (Figure S2). Although the approximate overlap of these midchain gaps is intriguing, they should be considered preliminary pending additional data from other studies, since our maps represent a modest number of patients and about half of our patients were not followed into their third or fourth decade.

Our population also gave us the opportunity to examine intrafamilial and interfamilial variability of this secondary feature of OI. These comparisons are limited to some extent by the broad age range of the participants and familial age gaps. Interfamilial variability was found at the *COL1A1* Gly589 residue. Four unrelated patients, two each with Types IV and III OI, had *COL1A1* Gly589Ser substitutions; mild HL was detected in one Types III and one Types IV, with onset at the age of 5 years in both cases, whereas the other Types III and IV participants had normal hearing when examined at the age of 3 and 15 years, respectively.

In *COL1A2*, there were three glycine residues in the amino‐quarter of the chain at which more than one participant had missense variants, involving both related and unrelated individuals. In a mother and her two daughters with Type IV OI caused by a Gly106Ser substitution, the mother had mild HL with onset at the age of 32, whereas the daughters had normal hearing at the ages of 16 and 6 years, leaving open the possibility of fourth decade onset in the children. A Gly238Ser substitution occurred in five individuals, two genetic half‐siblings with Type IV OI as well as two Type III and one Type IV OI unrelated participants. One half‐sibling had mild HL, as did one of the Type III OI participants, with onset at the ages of 10 and 3 years, respectively, whereas the second half‐sibling and unrelated Types III and IV participants had normal hearing when examined at the ages of 32, 17, and 15 years, respectively. Finally, Gly247Ser missense was found in three participants, a father and son with Type IV OI and an unrelated male with severe Type III OI. Both the Type III OI individual and the Type IV OI father had mild HL with onset at the ages of 17 and 11 years, respectively, whereas the Type IV OI son had normal hearing at age 14.

Thus, of the eight participants who share a collagen variant with at least one other participant and have normal hearing at last exam, four have normal hearing in the decade older than the corresponding participant with HL (normal hearing in second and fourth decades of life vs. first decade HL), whereas four participants with normal hearing are not yet old enough to be confident that HL will not develop at the older age of onset in their corresponding HL participant.

#### 3.3.2. Degree of HL, Type of HL, and Progression

For participants with variants in *COL1A1* and HL, the majority were mild and stable 67% (*n* = 14/21) but HL progressed in 33% to moderate (*n* = 2/7), severe (*n* = 4/7), or profound (*n* = 1/7). In the group with progression, all HL began as CHL but developed into MHL in five individuals (Figure 1). In the subset with stable mild HL, the majority of participants (*n* = 10/14) had CHL, with 4/10 individuals exhibiting SNHL at discreet visits. SNHL with no history of CHL was seen in 2/14 individuals, and HL type could not be determined in 2/14 (Table 2; Figure S3).

For participants with variants in *COL1A2* and HL, the degree of HL remained mild for all participants except one (*n* = 17/18), who had stable moderate HL in one ear. HL was conductive for the majority of participants (*n* = 13/18), with 4/13 individuals showing SNHL at discreet visits. One individual had SNHL with no history of CHL, and HL type could not be determined in 4/18 (Figure S3).

#### 3.3.3. Onset of HL

For both *COL1A1* and *COL1A2* variants, onset of HL was most common in the first decade (*n* = 16/21, 76%; *n* = 12/18, 67%, respectively). Further, a substantial proportion of HL onset occurred by 5 years of age, with early loss in 48% (*n* = 10/21) for *COL1A1* and 33% (*n* = 6/18) for *COL1A2* variants (Figure 2B).

### 3.4. HL Progression Is Greatest for Type III OI Participants With *COL1A1* Variants

PTA was modeled as a function of OI type, mutated gene, and visit to explore interactions between these factors. For 4F‐PTA, this revealed a significant interaction between visit and Type III OI (*β* = −0.028, 95% CI: [–0.044, –0.012], *p* = 0.0007), as well as a three‐way interaction between visit, genotype (*COL1A1*), and Type III OI (*β* = 0.053, 95% CI: [0.034, 0.072], *p* = 5.2*E* − 08), indicating that the rate of HL progression over time varied significantly across genotype‐OI type groups (Table S4).

Post hoc contrasts revealed that individuals with *COL1A1* variants exhibited significantly greater HL (4F‐PTA) than those with *COL1A2*, both in OI Type III (*β* = 0.35, 95% CI: [0.18, 0.52], *p* = 0.0001) and Type IV (*β* = 0.20, 95% CI: [0.0052, 0.40], *p* = 0.044). The genotype effect was more pronounced in Type III. In contrast, no significant differences in 4F‐PTA were observed between OI types within either genotype background (all *p* ≥ 0.38). Similar patterns of interaction and post hoc contrasts were observed in both LF‐PTA and HF‐PTA models, reinforcing the genotype– and OI Type–specific differences in hearing trajectory identified in 4F‐PTA analysis (Table S2).

Hearing trajectory revealed a significant increase in 4F‐PTA over time for individuals with *COL1A1* variants, with stronger HL progression in OI Type III (*β* = 0.038, 95% CI: [0.032, 0.044], *p* < 0.0001) compared with OI Type IV (*β* = 0.013, 95% CI: [0.0050, 0.021], *p* = 0.0014). Notably, individuals with *COL1A2* variants and OI Type III exhibited a significant negative slope (*β* = −0.023, 95% CI: [−0.038, −0.0083], *p* = 0.0023), suggesting potential stabilization or slight improvement in this subgroup (Figure 2C).

Breakpoints, defined as the age at which HL trajectory changes or begins to accelerate, were estimated separately for 4F‐PTA, LF‐PTA, and HF‐PTA. The mean estimated breakpoint for HL progression was age 13.5 specifically, for 4F‐PTA it was 13.0 ± 4.9 years, for LF‐PTA it was 13.7 ± 4.1 years, and for HF‐PTA it was 13.7 ± 2.7 years. The earliest breakpoints observed across all ears were 6.2 years for 4F‐PTA, 8.5 years for LF‐PTA, and 8.7 years for HF‐PTA, indicating that HL progression may begin as early as the first decade.

## 4. Discussion

In this paper, we have sought to identify patterns in OI‐related HL (type, degree, age of onset, and progression) as well as association of those features with OI skeletal phenotype (Sillence type) and causative gene variants. To do this, we have utilized the extraordinary longitudinal data from protocol participants with OI Type III/IV who were followed from childhood. All participants have a heterozygous causative variant in *COL1A1* or *COL1A2*, most of which result in a glycine substitution and a small number with skipping of a single exon or a small deletion. The most significant of the novel findings resulting from this analysis is an association of progression to more severe HL with *COL1A1* variants, particularly in participants who also have OI Type III.

The significant advantages of multidecade longitudinal data also allowed our analysis to amplify recent cross‐sectional reports, most notably the prevalence of early onset OI‐related HL in the first decade of life. This contrasts with earlier reports that established the orthodoxy that OI‐related HL had its onset in the second and third decades. However, our pediatric emphasis limits determination of adult‐onset HL in more recently enrolled patients, which warrants a complete characterization of HL findings over the lifespan.

In our longitudinal cohort, more than half of participants had at least mild HL, and the prevalence of HL in OI Type III was significantly greater (1.6x) than in OI Type IV. Almost all HL began as CHL and remained mild and stable in most OI Type III and OI Type IV participants over time, with about one‐quarter of those with CHL transitioning to MHL. When HL progressed to moderate or greater, the majority (67%), but not all, transitioned to MHL. This is a greater extent of HL than reported from the North American OI BBRC, with HL in about one‐quarter each of OI Types I, III, and IV [9]. The European collaboration had a strong focus on OI Type I (83.7% of participants) with an overall mean age of evaluation of 30 years. They reported an overall HL prevalence of 48.4%, with almost all converting to MHL or SNHL by the fifth decade [18]. An earlier study of a Finnish OI adult population, again with OI Type I most common, found HL in 57.9% of the patients, beginning in the second to fourth decades and most often MHL [6]. Thus, our study detected a higher percentage of HL among OI Type III/IV than the BBRC study, more in line with European studies. Given that the European studies have a majority composition of Type I OI, our data support a similar overall incidence of HL resulting from both changes in collagen primary sequence and insufficiency of normal collagen. In our study, the incidence of conversion of CHL to SNHL is low and MHL only occurs in the subgroup with progression to more severe HL. The low SNHL conversion in our group may be related to age; our cohort was predominantly under the age of 40 years, whereas other study populations skew older (40% of BBRC population over age 40 years) [6, 9]. Kuurila et al. [6] reported CHL limited to patients in the first three decades, with prevalence of SNHL increasing with age. In the BBRC report, the transition to MHL or SNHL occurs predominantly in OI Types I and III after the three decade and affects about half of these patients overall [9].

It had become “dogma” that OI‐related HL had its onset late in the second and mostly in the third decade of life. In line with these reports, our protocol initially undertook repeated audiological evaluation of children with the goal of finding features predictive of later HL development. Instead, we demonstrated onset of HL in the first decade of life in the majority of our OI Type III/IV population and before school‐age in 46% of OI Type III and 33% of OI Type IV, including among the group with progressive HL. There had been hints of early HL in other studies, likely limited by availability of children and predominance of OI Type I in study groups. In the Finnish pediatric studies, only 2 of 45 children were found with HL, both with OI Type IV, at the ages of 11 and 15 years [12]. From the European study [18] of 184 OI patients, 83.7% with OI Type I, a median age of onset of 20 years (range 5–60 years) was reported. At the younger end of this group, HL was detected in the first and second decades of life in 6.3% (*n* = 2/32 ears) and 34.1% (*n* = 30/88 ears), respectively [18]. The more recent cross‐sectional BBRC study detected HL in the first decade among approximately one‐fifth of OI Type III ears but not in OI Type IV, where onset occurred among 10% in the second decade [9]. The availability of longitudinal data likely contributed to the greater sensitivity of detection of early loss in our study, which was also drawn from across North America.

In addition, it is interesting to compare secondary features of OI Types III/IV, with heterozygous alterations in collagen protein sequence, with OI Type I, with insufficiency of normal type I collagen. There is a distinction in pathogenic mechanism and bone matrix composition between OI caused by haploinsufficiency variants and missense variants. In OI, haploinsufficiency refers to heterozygous variants in (almost always) *COL1A1* that result in a lack of stable alpha chain protein synthesis by the mutant allele (generally stop codons or PTCs from out‐of‐frame splicing). The patient thus synthesizes half the normal total amount of the alpha1 chain, and all the alpha1 chain that is synthesized has normal primary structure. At the matrix level, the amount of collagen is reduced and the pathology is due to insufficiency of the heterotrimeric Type I collagen molecule (*α*1(I)_2,_ *α*2(I)_1_). Individuals whose OI results from this type of mutation have the mildest form of OI, Type I OI. Many of the previous audiology studies were dominated by adults with Type I OI, representing only the clinical consequences of that pathology.

Mild HL in the first decade was reported among 19% of individuals with OI Type I without molecular confirmation [10]. The Finnish, European and BBRC studies all found that OI Type I populations had HL onset in the second to fourth decades, with the percentage of affected individuals increasing over time, supporting later development of HL in haploinsufficiency [6, 9, 18].

The availability of longitudinal data in a fully genotyped study population also yielded insights into genetic features among those with heterozygous variants in their Type I collagen primary protein sequence. Heterozygous single amino acid substitutions (almost always Gly substitutions in the first position of the Gly–X–Y collagen trimer) have a dominant negative pathologic mechanism, rather than a loss‐of‐function. The alpha chains containing the altered primary sequence are synthesized, incorporated into collagen heterotrimer in the cell, secreted and incorporated into extracellular matrix. For missense variants in one *COL1A1* allele, 75% of Type I collagen is expected to contain at least one chain with abnormal primary structure, whereas for missense variants in one *COL1A2* allele, 50% of Type I collagen is expected to contain a variant alpha chain. In general, missense variants are more deleterious to bone function than a reduced quantity of normal matrix. Hence, these missense variants with dominant negative pathology cause Types III OI, the severe progressive deforming type, and IV OI, which is moderately severe. The missense variants act by altering normal intracellular, cell‐matrix, matrix, and bone tissue functions. All patients in our study fall into this dominant negative category of OI pathology.

Genotype–phenotype correlations for skeletal aspects of OI have shown a complex mix of features—the proportion of Gly substitutions causing OI Type II, the perinatal lethal form, is greater for *COL1A1* than *COL1A2* variants, but there is not a straightforward association [30]. One possible explanation is the stoichiometry of Type I collagen heterotrimers, with two *α*1(I) and one *α*2(I) chains. Also, alpha chain functions differ, with *α*1(I) being especially critical to trimer stability and interaction with matrix molecules through a major ligand binding region, whereas *α*2(I) interactions along the chain involve matrix proteoglycans. These explanations support productive research but are not sufficient for clinically relevant predictions for patients. Here, we find that HL is associated with heterozygous variants altering the collagen primary proteins sequence (Gly substitutions, splicing, deletions) in either alpha chains, and that these variants causing HL are located along the helical region and even into the C‐propeptide (Figure 3). There is an intriguing midhelical gap in HL, which awaits reports of additional variants associated with HL from other studies to advance or undermine its functional status.

Our genetic data yielded two additional features. First, when looking at the interfamilial and intrafamilial concordance of HL outcome, we find the same phenotypic variability for HL as is found for skeletal features, with some variants having consistent outcomes and others having a variable outcome, pointing to modifying genetic factors yet to be identified. This variability was previously noted in the European study [18]. Second, and importantly, we find that, among individuals with heterozygous collagen missense variants as the cause of their OI, progression of HL beyond stable mild loss is limited to *COL1A1* mutations. The progressive HL begins as CHL and generally converts to MHL. Moreover, statistical analysis of interactions between HL, gene, and OI type reveals that progression is significantly associated with *COL1A1* variants causing OI Type III. This statistical preponderance of HL progression supports its association with greater overall OI severity, although noncollagen modifying factors are still likely to be contributory. An association of lower areal lumbar vertebral DXA z‐scores in OI patients with HL versus OI patients without HL was reported by Swinnen et al. in a genetically verified European OI group with 80% haploinsufficiency (32.7% normal hearing) and 20% collagen heterozygous missense variants (43% normal hearing) [8]. Since all haploinsufficiency mutations lead to the same outcome at the collagen protein level, undetermined modifying factors are again implied. The Finnish study found no association of HL with collagen alpha chain, but the predominantly Type I OI population and lack of longitudinal data did not allow examination of progression for individuals [7]. The BBRC study found more HL in Type III than IV OI but did not report collagen variants [9].

The data presented here extended the prevalence and severity of OI‐related HL to the molecular and genetic level but does not clarify the mechanism of its variability. At a molecular (biochemical) level, our data on HL progression in patients with *COL1A1* variants are consistent with collagen stoichiometry. At the bone tissue level, CHL and MHL could result from bone fragility and bone remodeling around the otic capsule due to abnormal collagen structure and bony changes in the ossicles and otic capsule, but these anomalies are also evidenced in individuals with no HL [31]. The substantial incidence of HL in OI Type I, in which there is insufficiency of normally structured collagen, shows that abnormal collagen structure is not necessary to HL. CHL and MHL in the OI population have often been attributed to otosclerosis and stapes footplate fixation [32] but in OI, there is a higher prevalence of footplate thickening and brittleness [33].

Early identification of HL allows individuals to seek treatment through amplification and accommodations at home, school, and work [34]. Late‐identified and untreated HL has multiple impacts on language development, education, employment, cognition, mental health, and overall quality of life across the lifespan [34]. Even children with mild HL are at risk for delays in speech and language development, listening comprehension, and academic achievement highlighted in current early detection guidance [35].

Pending further understanding of OI‐related HL mechanisms, the novel findings in this paper can be used empirically to update clinical guidance. The OI care team should prioritize early detection of HL in preschool years, establishing a baseline by 9 months of age, in support of early intervention [34]. Hearing monitoring in OI Types III and IV should continue over the lifetime, with more frequent testing for individuals with *COL1A1* variants and OI Type III, whereas individuals with *COL1A2* may require less frequent monitoring.

## Author Contributions

Conceptualization and methodology: J.A.C., J.C.M., and G.L.P. Data curation: E.F.E., J.A.C., A.T., H.C., S.T., J.C.M., and G.L.P. Formal analysis: J.A.C., A.T., H.C., S.T., J.C.M., and G.L.P. Investigation: J.A.C., C.C.B., C.Z., J.C., T.T.W., L.N.A., S.T., E.F.E., A.D., J.C.M. Project administration: S.T. and A.D. Supervision: J.C.M. and G.L.P. Writing—original draft: J.A.C., A.T., H.C., J.C.M., and G.L.P. Writing—review and editing: remaining authors. J.C.M. and G.L.P. contributed equally to this work.

## Funding

This study was supported by Eunice Kennedy Shriver National Institute of Child Health and Human Development (10.13039/100009633; ZIA HD008830‐16) and National Institute on Deafness and Other Communication Disorders (10.13039/100000055; ZIA DC000064).

## Disclosure

The contributions of the NIH authors are considered works of the United States Government. The findings and conclusions presented in this paper are those of the authors and do not necessarily reflect the views of the NIH or the US Department of Health and Human Services.

## Ethics Statement

The Natural History of the Collagen‐Related Disorder Osteogenesis Imperfecta and the Genotype–Phenotype Correlation protocol was approved by the Institutional Review Board of the National Institute of Child Health and Human Development (NICHD). The trial was conducted in accordance with the principles set out in the Declaration of Helsinki. Written informed consent was provided by all participants or parent(s)/legal guardian(s) as required by the IRB.

## Conflicts of Interest

The authors declare no conflicts of interest.

## Supporting Information

Additional supporting information can be found online in the Supporting Information section.

## Supporting information


**Supporting Information 1** Table S1: Operational definitions of pure tone averages, degree, and type of hearing loss.

**Supporting Information 2** Table S2: Post hoc comparisons and visit slopes of hearing by genotype and OI type (4F‐PTA).

**Supporting Information 3** Table S3: Final decade of completed audiological measures by gene and decade.

**Supporting Information 4** Table S4: PTA as a function of OI type, gene variant, and visit.

**Supporting Information 5** Figure S1: Normal hearing, degree of hearing loss and progression by OI type. All ears (individual lines) with normal hearing (WNL = within normal limits; gray circles), hearing loss (horizontal dashed line represent degree of hearing loss designation; > 20 dBHL is hearing loss), and type (CHL = blue triangle, MHL = yellow inverted triangle, and SNHL = red square) indicated as a function of age (years). Each row represents LF‐, 4F‐, and HF‐PTA with columns noting normal hearing, stable or progressive hearing loss, by OI Type III or IV. Bone conduction not measured indicated by “x,” and unfilled circles represent cases unable to be categorized by hearing loss type. Refer to Figure 1 for representation of only ears with hearing loss.

**Supporting Information 6** Figure S2: OI Type IV hearing by PTA for quartiles. All ears (individual lines) representing degree and type of hearing loss by (rows) LF‐, 4F‐, HF‐PTA and age (years) sorted by quartile (columns; Q1, Q2, Q3, and Q4). One individual had a bilateral high frequency hearing loss in Q3 in the fourth decade of life, but hearing was not measured in the third decade. Same colors and representation of symbols as Figure 1.

**Supporting Information 7** Figure S3: Hearing by gene variant and OI type. All ears (individual lines) represent degree and type of hearing loss by age (years). Columns represent gene variant and rows represent LF‐, 4F‐, and HF‐PTA. Normal hearing, stable hearing, and progressive hearing loss are shown together. Same colors and representation of symbols as Figure 1.

## Data Availability

Aggregated, deidentified data can be made available upon reasonable request to the corresponding author J.C.M.
