# Supplementary material for: Type III Osteogenesis Imperfecta and COL1A1 Pathological Variants Are Associated With Higher Incidence and Progression of Hearing Loss
Source: Hum Mutat. 2026 Aug 21;2026:6245371. doi: 10.1155/humu/6245371 (PMC13498850; doi:10.1155/humu/6245371)
Supplement: Supplementary file 1 [file HUMU-2026-6245371-s001.docx]

| **Supplemental Table 1**. Operational Definitions of Pure Tone Averages, Degree, and Type of Hearing Loss | | | | | |
| --- | --- | --- | --- | --- | --- |
| **Pure Tone Average (PTA)** | | **Degree of Hearing Loss** | | **Type of Hearing Loss** | |
| LF | (0.25/0.5 kHz) | Mild | >20 and ≤40 dB HL | Subclinical | AC ≤20 dB HL and ABG >10 dB |
| 4F | (0.5/1/2/4 kHz) | Moderate | >40 and ≤70 dB HL | Conductive | AC >20 dB HL, BC ≤20 dB HL, and ABG >10 dB |
| HF | (4/8 kHz) | Severe | >70 and ≤95 dB HL | Mixed | AC >20 dB HL, BC >20 dB HL, and ABG >10 dB |
|  | | Profound | >95 dB HL | Sensorineural | AC >20 dB HL and ABG ≤10 dB |
|  | |  | | Uncategorized | HF hearing loss could not be estimated using 4 kHz BC |
|  | |  | | Unknown | BC thresholds were not available |

*Key: LF=low frequency; 4F=4 frequency; HF=high frequency; dB HL=decibels in hearing level; AC=air conduction; BC=bone conduction; ABG=air bone gap.*
