# Supplementary material for: Type III Osteogenesis Imperfecta and COL1A1 Pathological Variants Are Associated With Higher Incidence and Progression of Hearing Loss
Source: Hum Mutat. 2026 Aug 21;2026:6245371. doi: 10.1155/humu/6245371 (PMC13498850; doi:10.1155/humu/6245371)
Supplement: Supplementary file 2 [file HUMU-2026-6245371-s002.docx]

| **Supplemental Table 2.** Post Hoc Comparisons and Visit Slopes of Hearing by Genotype and OI Type (4F-PTA) | | | | | | |
| --- | --- | --- | --- | --- | --- | --- |
| **Contrast/Slope** | **Estimate (β)** | **SE** | **df** | **t** | **95% CI** | **p-value** |
| *COL1A1* vs *COL1A2* [Type III] | 0.35 | 0.084 | 57.4 | 4.15 | [0.18, 0.52] | **0.0001** |
| *COL1A1* vs *COL1A2* [Type IV] | 0.2 | 0.097 | 46.8 | 2.07 | [0.0052, 0.40] | **0.044** |
| Type III vs Type IV [*COL1A1*] | 0.066 | 0.086 | 46.9 | 0.77 | [-0.11, 0.24] | 0.45 |
| Type III vs Type IV [*COL1A2*] | -0.082 | 0.093 | 54.6 | -0.88 | [-0.27, 0.11] | 0.38 |
| Visit slope [*COL1A1*, Type III] | 0.038 | 0.0033 | 719 | 11.65 | [0.032, 0.044] | **<0.0001** |
| Visit slope [*COL1A1*, Type IV] | 0.013 | 0.004 | 715 | 3.21 | [0.0050, 0.021] | **0.0014** |
| Visit slope [*COL1A2*, Type III] | -0.023 | 0.0075 | 723 | -3.06 | [-0.038, -0.0083] | **0.0023** |
| Visit slope [*COL1A2*, Type IV] | 0.0048 | 0.0033 | 715 | 1.46 | [-0.0017, 0.011] | 0.14 |
