# Supplementary material for: Type III Osteogenesis Imperfecta and COL1A1 Pathological Variants Are Associated With Higher Incidence and Progression of Hearing Loss
Source: Hum Mutat. 2026 Aug 21;2026:6245371. doi: 10.1155/humu/6245371 (PMC13498850; doi:10.1155/humu/6245371)
Supplement: Supplementary file 3 [file HUMU-2026-6245371-s003.docx]

| **Supplemental Table 3.** Final Decade of Completed Audiological Measures by Gene and Decade | | | | | |
| --- | --- | --- | --- | --- | --- |
| **Gene** | **Decade 1** | **Decade 2** | **Decade 3** | **Decade 4** | **Decade 5** |
| ***COL1A1*** | 22% | 26.8% | 36.6% | 14.6% | 0% |
| ***COL1A2*** | 15.6% | 43.8% | 21.9% | 15.6% | 3.1% |
