# Supplementary material for: Type III Osteogenesis Imperfecta and COL1A1 Pathological Variants Are Associated With Higher Incidence and Progression of Hearing Loss
Source: Hum Mutat. 2026 Aug 21;2026:6245371. doi: 10.1155/humu/6245371 (PMC13498850; doi:10.1155/humu/6245371)
Supplement: Supplementary file 4 [file HUMU-2026-6245371-s004.docx]

| **Supplemental Table 4.** PTA as a function of OI Type, Gene Variant, and Visit | | | | | |  |
| --- | --- | --- | --- | --- | --- | --- |
|  | **Estimate (β)** | **SE** | **df** | **t** | **95% CI** | **p-value** |
| **(Intercept)** | 0.84 | 0.089 | 47.7 | 9.39 | [0.66, 1.02] | **2.0E-12** |
| **Sex** |  |  |  |  |  |  |
| **Male** | reference |  |  |  |  |  |
| **Female** | 0.099 | 0.059 | 41.7 | 1.68 | [-0.019, 0.22] | 0.10 |
| **Age** | 0.019 | 0.0056 | 45.1 | 3.37 | [0.0076, 0.030] | **0.0015** |
| **Visit** | 0.0048 | 0.0033 | 718.6 | 1.47 | -0.0016, 0.011] | 0.14 |
| **Genotype** |  |  |  |  |  |  |
| ***COL1A2*** | reference |  |  |  |  |  |
| ***COL1A1*** | 0.15 | 0.096 | 52.3 | 1.52 | [-0.046, 0.34] | 0.13 |
| **OI Type** |  |  |  |  |  |  |
| **Type IV** | reference |  |  |  |  |  |
| **Type III** | 0.11 | 0.092 | 59.9 | 1.20 | [-0.073, 0.29] | 0.23 |
| **Visit x Genotype *COL1A1*** | 0.0080 | 0.0051 | 721.8 | 1.55 | [-0.0021, 0.018] | 0.12 |
| **Visit x OI Type III** | -0.028 | 0.0082 | 724.1 | -3.42 | [-0.044, -0.012] | **6.7E-04** |
| **Genotype *COL1A1* x OI Type III** | -0.22 | 0.12 | 57.8 | -1.75 | [-0.47, 0.031] | 0.086 |
| **Visit x Genotype *COL1A1* x OI Type III** | 0.053 | 0.0096 | 723.5 | 5.50 | [0.034, 0.072] | **5.2E-08** |

Log (4F-PTA) ~ Sex + Age + Visit x Genotype x OI Type + (1| Subject/Ear); Observation: 764; Subject: 41; Overall model evaluation: *Akaike Information Criterion* = -106.3, *Bayesian Information Criterion* = -46.0, r^2^ _conditional_ = 0.60, r^2^ _marginal_ = 0.27, ICC = 0.46.
