# Supplementary material for: Type III Osteogenesis Imperfecta and COL1A1 Pathological Variants Are Associated With Higher Incidence and Progression of Hearing Loss
Source: Hum Mutat. 2026 Aug 21;2026:6245371. doi: 10.1155/humu/6245371 (PMC13498850; doi:10.1155/humu/6245371)
Supplement: Supplementary file 7 [file HUMU-2026-6245371-s007.docx]

**Supplemental Figure 3**
